# ECG Changes during Adult Life in Fabry Disease: Results from a Large Longitudinal Cohort Study

**DOI:** 10.3390/diagnostics13030354

**Published:** 2023-01-18

**Authors:** Mohamed El Sayed, Pieter G. Postema, Mareen Datema, Laura van Dussen, Jan A. Kors, Cato C. ter Haar, Hidde Bleijendaal, Henrike Galenkamp, Bert-Jan H. van den Born, Carla E. M. Hollak, Mirjam Langeveld

**Affiliations:** 1 Department of Internal Medicine, Division of Endocrinology and Metabolism, Amsterdam UMC Location University of Amsterdam, Meibergdreef 9, 1105 AZ Amsterdam, The Netherlands; 2 Amsterdam Gastroenterology Endocrinology and Metabolism, Inborn Errors of Metabolism, 1105 AZ Amsterdam, The Netherlands; 3 Department of Cardiology, Heart Center, Amsterdam UMC Location University of Amsterdam, Meibergdreef 9, 1105 AZ Amsterdam, The Netherlands; 4 Amsterdam Cardiovascular Sciences, Heart Failure & Arrhythmias, 1105 AZ Amsterdam, The Netherlands; 5 Department of Medical Informatics, Erasmus MC, University Medical Center Rotterdam, 3015 GD Rotterdam, The Netherlands; 6 Department of Biostatistics & Bioinformatics, Amsterdam UMC Location University of Amsterdam, Meibergdreef 9, 1105 AZ Amsterdam, The Netherlands; 7 Department of Public and Occupational Health, Amsterdam UMC, Location AMC, University of Amsterdam, Meibergdreef 9, 1105 AZ Amsterdam, The Netherlands; 8 Amsterdam Public Health, Health Behaviors and Chronic Diseases, 1105 AZ Amsterdam, The Netherlands; 9 Department of Vascular Medicine, Amsterdam UMC Location University of Amsterdam, Meibergdreef 9, 1105 AZ Amsterdam, The Netherlands; 10 Amsterdam Cardiovascular Sciences, Atherosclerosis & Ischemic Syndromes, 1105 AZ Amsterdam, The Netherlands

**Keywords:** Fabry disease, Fabry cardiomyopathy, Electrocardiogram (ECG), treatment evaluation

## Abstract

**Background:** Fabry disease (FD) is an X-linked, lysosomal storage disorder leading to severe cardiomyopathy in a significant proportion of patients. To identify ECG markers that reflect early cardiac involvement and disease progression, we conducted a long term retrospective study in a large cohort of FD patients. **Methods:** A total of 1995 ECGs from 133 patients with classical FD (64% females, 80% treated with enzyme replacement therapy), spanning 20 years of follow-up, were compared to ECGs from 3893 apparently healthy individuals. Generalized linear mixed models were used to evaluate the effect of age, FD and sex on: P-wave duration, PR-interval, QRS-duration, QTc, Cornell index, spatial QRS-T angle and frontal QRS-axis. Regression slopes and absolute values for each parameter were compared between FD patients and control subjects. **Results:** At a younger age (<40 years), the Cornell index was higher and frontal QRS-axis more negative in FD patients compared to controls (*p* < 0.05). For the other ECG parameters, the rate of change, more than the absolute value, was greater in FD patients compared to controls (*p* < 0.05). From the fifth decade (men) or sixth (women) onwards, absolute values for P-wave duration, QRS-duration, QTc and spatial QRS-T angle were longer and higher in FD patients compared to control subjects. **Conclusions:** ECG abnormalities indicative of FD are age and sex dependent. Tracking the rate of change in ECG parameters could be a good way to detect disease progression, guiding treatment initiation. Moreover, monitoring ECG changes in FD can be used to evaluate the effectiveness of treatment.

## 1. Introduction

Fabry disease (FD) is an X-linked lysosomal storage disease with slowly progressive and highly variable clinical expression. The disorder is caused by mutations in the galactosidase alpha (GLA) gene, leading to reduced activity of the lysosomal enzyme alpha-galactosidase A. The enzymes’ substrate globotriaosylceramide (Gb3) and its derivates accumulate in various tissues and organs, including the heart [1,2]. Over the decades, lysosomal dysfunction, disturbed autophagy, inflammatory and fibrotic changes eventually lead to permanent cardiac damage [3,4,5,6]. Initially, signs of cardiac involvement may be subtle, such as a low native T1 value on cardiac magnetic resonance imaging (CMR) and a short PR-interval on ECG. At that early stage there are most often no clinical symptoms of cardiac disease and overall cardiac function on echocardiography and CMR is normal [7,8]. At later disease stages, conduction abnormalities, overt left ventricular hypertrophy (LVH), myocardial fibrosis and eventually symptomatic cardiac disease (heart failure, arrhythmias and sudden cardiac death) occurs [9,10,11,12].

Since FD is an X-linked disorder the disease is generally more severe in men. The disease can be classified into an early onset, classical, and a later-onset, non-classical phenotype [9,10,13]. A recently conducted observational longitudinal cohort study in 213 FD patients confirmed the heterogeneity of cardiac disease manifestations in FD [14]. Male patients with classical FD (cFD) over the age of 45 years, invariably suffered a cardiovascular event. In contrast, only a subset of females with cFD developed cardiovascular events and with a highly variable age of onset [14]. For male patients with cFD there is no debate about the need for Fabry specific treatment and recent studies suggest that early initiation (specifically of enzyme replacement therapy (ERT) has a better effect on suppressing disease progression) [15,16]. For women with cFD, there is much more uncertainty about the need for treatment of an individual patient (since not all patients will develop complications) and even more about the optimal timing of treatment initiation. Thus, there is a need to identify female patients at risk of symptomatic cardiac disease (and thus in need of intensive monitoring) versus those who are unlikely to develop major cardiac complications.

LVH on CMR is a commonly used clinical marker for the presence of cardiac manifestation in FD and thus the need to initiate therapy [15]. However, in those that develop LVH, this usually occurs in later stages of the disease and some female patients develop cardiac FD complications in the absence of LVH [10,17]. Hence, identification of biomarkers (other than age, sex and left ventricular mass) that can reliably detect cardiac involvement at an earlier, asymptomatic, disease stage is important to be able to limit further progression of cardiac disease in FD patients. ECG parameters might be suitable to track this progression. 

Previous cross-sectional and longitudinal studies with a relatively small sample size show that early ECG abnormalities in FD patients include a short PR-interval and bradycardia [18,19]. A long P-wave duration, prolonged QRS-duration, QTc, high QRS-amplitude, T-wave inversion and left frontal QRS- axis deviation may occur in later disease stages [19,20,21]. There are no longitudinal studies to show electrophysiological development over time in different patient groups (e.g., men versus women with FD) and how this differs from age related changes in ECG parameters in healthy individuals. This knowledge is needed to guide the timing of treatment initiation (especially in women). However, to also be able to detect the effect of (new) FD treatments on cardiac disease progression, since overt clinical complications take decades to develop [22,23], far surpassing the duration of clinical trials.

We thus conducted a retrospective study in the FD patient cohort under follow-up at the Amsterdam University Medical Centres (AUMC), to establish the course of electrophysiological parameters in male and female FD patients, to compare them to apparently healthy control subjects and to study their relationship to left ventricular mass and the presence of fibrosis on CMR. This study is unique in terms of both sample size and length of systematic ECG follow-up of FD patients.

**Primary aims of the study are:**

1. Describing the evolution of alterations in ECG parameters in patients with classical FD and comparing these features to those of an apparently healthy control group;
2. Comparing the evolution of ECG alterations in men versus women with classical FD.

**The secondary aim is:**

3. Investigating the relationship between ECG features and left ventricular mass and the presence of late gadolinium enhancement on CMR in classical FD patients.

## 2. Methods

### 2.1. Study Population and Design

#### 2.1.1. FD Patients

This retrospective cohort study was conducted at the FD centre of excellence in the Netherlands (AUMC, location University of Amsterdam). All available ECGs from adult (≥18 years) patients with classical FD obtained between February 1996 and July 2018 were included. The ECGs were obtained as part of the routine clinical follow-up (outpatient clinical visits are every 6 to 12 months, depending on age, sex and treatment status of the patient) or during hospital admissions. All patients of whom ECGs were included had a definite diagnosis of classical FD. The diagnosis and phenotype assignment were based on alpha-galactosidase A in leucocytes, family history (for women), classical FD symptoms and the levels of a deacylated form of Gb3 (Globotriaosylsphingosine (LysoGb3)) (Supplemental Figure S1) [9,10,13].

#### 2.1.2. Control Group

ECGs of apparently healthy subjects of Dutch origin were collected between January 2011 and November 2015 as part of the baseline data collection of the ‘HELIUS’ study (Healthy Life in an Urban Setting—a large prospective cohort study in Amsterdam, The Netherlands) [24]. HELIUS is a multi-ethnic study, including roughly equally sized groups of Surinamese (South-Asian and African), Ghanaian, Turkish, Moroccan and Dutch origin participants, and was designed to study the (causes of the) unequal burden of disease across ethnic groups. To increase comparability with the FD cohort, of which the vast majority is of Dutch origin, only HELIUS participants with Dutch origin were selected as controls. In addition, age was restricted to 18–65 for men, and 18–71 for women, since this was the age range in FD patients. Baseline measurements of HELIUS included a single ECG during a physical examination at the research location, collection of biological samples and questionnaires [24,25]. Individuals with and without cardiovascular risk factors (including smoking, hypertension, obesity, diabetes mellitus and the use of anti-lipaemics, full list and definitions can be found in Table 1) were included, but they had to be free from apparent cardiovascular disease (myocardial infarction or stroke) as determined by self-report, medication use and ECG evaluation. We chose a control group in which cardiovascular risk factors were present since these same risk factors were also present in a significant number of the included FD patients (Table 1).

#### 2.1.3. Ethics Approval

This work was conducted following the Declaration of Helsinki [26]. Due to the retrospective nature of this study, the need for informed consent for the use of data from the FD patients was waived by the Medical Ethics Committee of the Amsterdam UMC. The HELIUS study was approved by the same Medical Ethics Committee and all participants provided written informed consent.

### 2.2. ECG Processing and Analysis

Standard 12-lead supine digital resting ECGs were recorded in FD patients and control subjects (GE MAC5500, 500 samples/s). The obtained ECGs were processed with the Modular ECG Analysis System (MEANS) program, which determines P-wave, QRS and T-wave onsets and offsets for all 12 leads together on one representative averaged beat [27]. All on- and offsets were manually checked and adjusted when needed. Thereafter, various ECG parameters were automatically computed, including P-wave duration, PR-interval, QRS-duration, QTc, QRS minimum and maximum amplitudes for each lead, Spatial QRS-T angle, Frontal QRS-axis and Frontal T-axis. In the case of ventricular pacing, all parameters were excluded from the analysis, and in the case of atrial fibrillation or atrial pacing, P-wave duration and PR-interval were selectively excluded from analysis.

### 2.3. Cardiovascular Risk Factors and Clinical Characteristics

For FD patients, cardiovascular risk factors (smoking, hypertension, obesity, diabetes mellitus and dyslipidemia, full list and definitions can be found in Table 1) were assessed at the first outpatient visit. With the exception of dyslipidemia, the same cardiovascular risk factors were surveyed in the control group. As an alternative to dyslipidemia evaluation in the control subjects, we reported the use of antilipaemics.

Data on estimated glomerular filtration rate (eGFR) and the presence of albuminuria were collected. For FD patients, these data were extracted from the clinical records for the date closest to the last included ECG (maximum 1 year earlier or later).

Renal function was estimated by calculating the estimated glomerular filtration rate (eGFR) using the CKD-EPI formula. Microalbuminuria in FD patients was defined as ≥30 mg albumin in collected 24 h urine samples. For the control subjects, microalbuminuria was defined as ≥20 mg/L albumin in a urine portion, as no 24-hour urine samples were available.

### 2.4. ECG and CMR Characteristics in FD Patients

To assess the relation between ECG parameters and cardiac imaging parameters, CMR data (left ventricular mass indexed to body surface area (LVMi) and the presence of late gadolinium enhancement (LGE) were extracted from the patients records for the date closest to the last included ECG (maximum 1 year earlier or later).

### 2.5. Cardiovascular Events in FD Patients

Cardiac events from birth to last outpatient clinic visit were recorded in FD patients (the majority of the patients included in the current study were also included in our recently published study on cardiovascular events in FD [14]). Recorded cardiac events were major cardiovascular events (MACE) (combined endpoint including cardiovascular death, heart failure hospitalization, sustained ventricular arrhythmias and myocardial infarction) (for definitions of these events see: Supplemental Table S3).

### 2.6. Statistical Analysis

R Studio (version 4.0.3) was used for statistical analysis. Data are presented as proportions or median and minimum/maximum ranges. Differences in the prevalence of cardiovascular risk factors between FD patients and the control group were tested by the 2 × 2 Fisher exact test. Generalized linear mixed-effect models (GLM) were used to evaluate the effect of age at the time of obtaining an ECG, type of study subject (FD patient or control subject) and sex on seven ECG parameters: P-wave duration, PR-interval, QRS-duration, QTc, Cornell Index (voltage sum of R in aVL and S in V3), spatial QRS-T angle and frontal QRS-axis. A random intercept and slope were introduced into all mixed models, taking inter-patient differences into account. Model assumptions were checked and met [28]. Due to the assumption that the effect of age on ECG parameters would be different between men and women, but also between FD patients and controls, we tested for three-way interactions (age * type of the study subject (FD patient or control subject) * sex) in all models. Subgroups were defined as: men with classical FD, women with classical FD, controls- men and controls- women. From the resulting model, regression lines per subgroup were obtained following the standard regression equation for a linear model: y = a + β*X, with the intercept (a) and slope (β) specified for each subgroup. Similarly, the differences between two given subgroups were calculated. The slopes of these regression equations (β per 10 years increase in age) were compared to study the difference in evolution of a given ECG parameter over time between subgroups.

In a GLM subanalysis, the untreated FD patients were excluded to investigate if there was a difference in ECG parameters’ increment between the ERT treated patients only and the complete study cohort, that also contains a minority of untreated FD patients. The frontal T-axis showed too much nonlinear variation for a valid GLM approach. For that reason, only the raw data on frontal T-axis over time were visually displayed in a polar plot.

The GLM assumes a linear change in ECG parameters over time, and is therefore unsuitable for making an accurate statement about the age at which an ECG parameter in FD patients start to deviate from those in control subjects. To study the timeframe in which ECG alterations occur in FD, the differences in absolute values for each ECG parameter between FD patients and the control subjects for each decade of adult life were assessed by a Wilcoxon rank test. For these comparisons, a Bonferroni adjusted *p*-value is displayed to correct for multiple testing. For this comparison of absolute values, only the last available ECG per patient per decade was selected to ensure that the influence of repeated measurements in single individuals was limited.

By using a Spearman correlation analysis, the correlation between the seven electrophysiological parameters on the last obtained ECG and LVMi on the corresponding CMR was evaluated. A Wilcoxon signed-rank was used to study if the absolute values of the parameters on the last ECG were different between patients with and without LGE on the corresponding CMR (LGE assesses the presence of myocardial fibrosis). We regarded a *p*-value ≤ 0.05 as statistically significant.

## 3. Results

### 3.1. Participants’ Characteristics

Serial ECGs of a total of 133 patients with classical FD (36% men and 64% women) were included averaging 15 ECGs per patient (range: 4–66), totaling 1995 ECGs. For the FD patients, the median age at last obtained ECG was 48 years (range: 19–82). The HELIUS cohort consisted of 3893 control subjects (43% men and 57% women) with a median age of 46 years (range: 18–71). Full description of the participants’ characteristics can be found in Table 1.

Eighty percent (106/133) of the included FD patients were treated with ERT, during a median period of seven years (range: 0–17). ERT initiation decisions were based on the presence of FD symptoms [29] or on recommendations by the European Fabry Working Group after these became available in 2015 [15]. See Supplemental Table S4 for the GLA mutations characteristics of the FD patients.

### 3.2. ECG Parameters

The reported conduction times—P-wave duration, PR-interval and QRS-duration—and represent structural modifications to the conduction system. Repolarisation problems are characterized by the QTc and frontal T-axis. Additionally, the Cornell index and frontal QRS-axis are indicative of anatomical alterations in the LV myocardium. Lastly, the relationship between myocardial depolarisation and repolarisation is well depicted by the spatial QRS-T angle. The modelled increment of each ECG parameter per 10 years was not different between the ERT treated patient group (N = 106) and the complete study cohort, which included 27 untreated patients (N = 133) (see Supplemental Table S1C,D). Thus, excluding the untreated patients did not alter the observed changes in the ECG parameters. For this reason, we report further results for the complete study cohort only.

**Figure 1:**
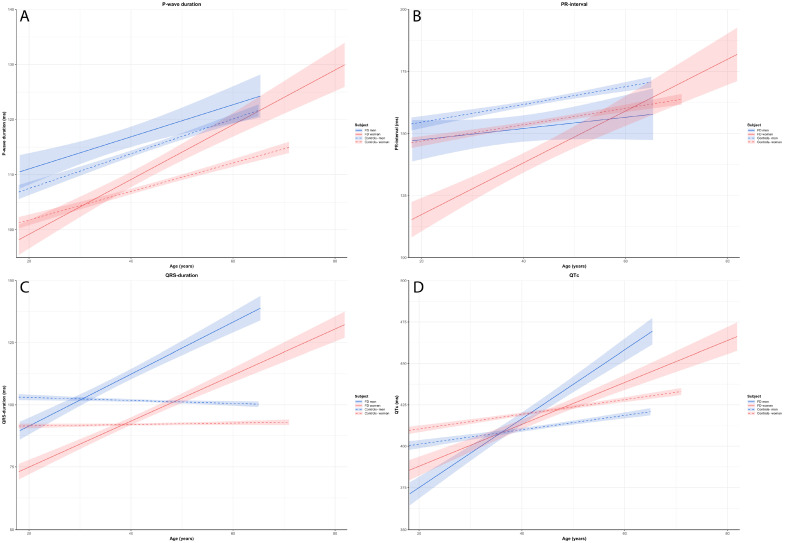
Based on the GLM, the estimated effect plots with the 95%-CI of (**A**) P-wave duration, (**B**) PR-interval, (**C**) QRS-duration and (**D**) QTc for each study participants.

**Figure 2:**
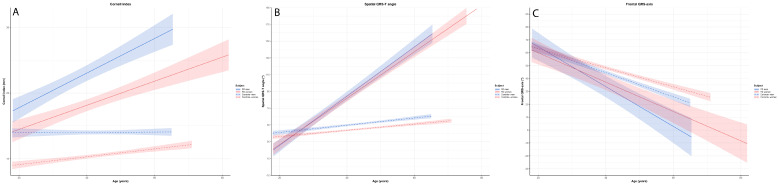
Based on the GLM, the estimated effect plots with the 95%-CI of (**A**) Cornell index, (**B**) Spatial QRS-T angle and (**C**) Frontal QRS-axis for each study participants subgroup.

**Figure 3:**
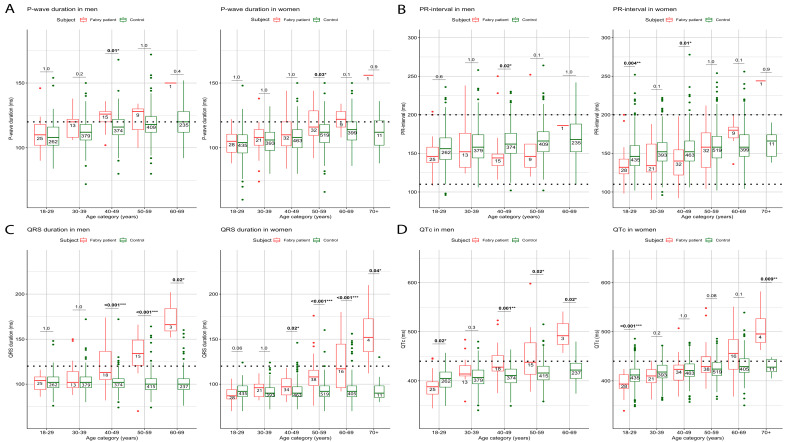
Boxplots of (**A**) P-wave duration, (**B**) PR-interval, (**C**) QRS-duration and (**D**) QTc per age decade in Fabry patients and controls. Numbers inside the boxes are the numbers of the analyzed ECGs. The last available ECG per FD patient per decade was selected to ensure that the influence of repeated measurements was limited. The horizontal lines represents the reference ranges of each ECG parameter, based on the literature. * p < 0.05, ** p < 0.01, *** p < 0.001.

**Figure 4:**
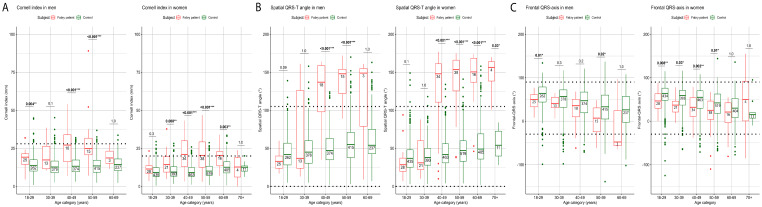
Boxplots of (**A**) Cornell index, (**B**) Spatial QRS-T angle and (**C**) Frontal QRS-axis per age decade in Fabry patients and controls. Numbers inside the boxes are the numbers of the analyzed ECGs. The last available ECG per FD patient per decade was selected to ensure that the influence of repeated measurements was limited. The horizontal lines represent the reference ranges of each ECG parameter, based on the literature. * *p* < 0.05, ** *p* < 0.01, *** *p* < 0.001.

The results of the GLM are presented in Table 2A,B and Supplemental Table S1. Figure 1 and Figure 2 display the modelled course of each ECG parameter, while raw longitudinal ECG data are presented in Supplemental Figures S2–S5. Boxplots and the descriptive statistics of the absolute values of ECG parameters per age decade are displayed in Figure 3 and Figure 4 and Supplemental Table S2, respectively.

#### 3.2.1. P-Wave Duration and PR-Interval

In all subgroups, P-wave duration increased significantly with increasing age. Women with FD showed a greater increment in P-wave duration with age compared to women controls (women: FD minus controls β = 2.4 ms per decade; 95%-CI 1.4–3.4) and compared to men with FD (FD: women minus men β = 2.1 ms per decade; 95%-CI 0.5–3.6). In men, the increase in P-wave duration with ageing was similar for FD patients and control subjects (Table 2A, Figure 1A). In patients with FD, the absolute values for P-wave duration were significantly longer in men between age 40–50 years and in women between age 50–60 years compared to control subjects. These differences were not significant in the decades thereafter, but the number of observations in these later decades was low (Figure 3A). P-wave duration showed median values above the normal range of 120 ms from 30–40 years in FD men and 50–60 years in FD women onwards (Supplemental Table S2, Figure 3A).

PR-interval significantly increased with ageing in both control groups and in women with FD, but not in men with FD (Table 2A). In women, PR-interval showed a significantly faster prolongation with age in women with FD as compared to controls (women: FD- controls β = 7.1 ms per decade; 95%-CI 4.5–9.7), whilst in men with FD vs. men controls this difference was not significant. In line with this observation, women with FD had a significantly faster prolongation with age compared to men with FD (FD: women-men β = 8.2 ms per decade; 95%-CI 4.1–12.3) (Table 2A, Figure 1B). PR-interval in women with FD was significantly lower compared to control subjects up to the age of 50 years. Notably, the PR-interval remained within the normal range of 120–200 ms [29] in FD patients in all age categories [30] (Figure 3B).

#### 3.2.2. QRS-Duration and QTc

QRS-duration showed a significant prolongation over time in patients with FD, in contrast to controls in whom QRS-duration was similar at all ages. This was reflected by a significant difference in the increment of QRS-duration in FD patients as compared to controls (men: FD minus controls: β = 11.1 ms per decade; 95%-CI 9.4–12.7, women: FD patients minus controls β = 9.0 ms per decade; 95%-CI 7.7–10.2) (Table 2A, Figure 1C). The increment in QRS- duration with ageing did not differ between men and women with FD (FD: men minus women β = 1.2 ms per decade; 95%-CI −0.8–3.2), but QRS-duration was significantly longer in male patients with FD compared to female patients during the entire follow-up period (*p* = 0.001) (Figure 1C). Up until 40 years, the absolute values for QRS-duration were not different in FD patients compared to control subjects, while after 40 years, QRS-duration became significantly longer both in male and female FD patients. The QRS-duration reached a threshold of 120 ms or higher between age 40–50 years in males with FD, whilst this point in FD females was reached between age 60–70 years (Supplemental Table S2, Figure 3C).

QTc showed significant prolongation with increasing age in all four subgroups, but progression was greater in FD patients (men: FD minus controls: β = 16.4 ms per decade; 95%-CI 13.6–19.3 and women: FD minus controls: β = 8.2 ms per decade; 95%-CI 6.0–10.5, respectively). The difference in QTc could not be explained by difference in heart rate between FD patients and the controls (Supplemental Figures S6 and S7). In addition, the increment in QTc with ageing was more pronounced in men with FD compared to women with FD (FD: men minus women: β = 8.2 ms per decade; 95%-CI 4.7–11.6) (Table 2A, Figure 1D). In accordance with the model, the differences in the absolute value of QTc between FD patients and controls became more pronounced throughout adult life. The QTc reached a threshold of 440 ms or higher between age 40–50 years in men with FD, while abnormal QTc values in women with FD were observed from 50 years of age onwards (Supplemental Table S2, Figure 3D).

#### 3.2.3. Cornell Index, Spatial QRS-T Angle and Frontal QRS-Axis

In patients with FD, Cornell index values showed a significant increase with ageing compared to controls where it remained stable (in men) or showed a less pronounced increase (in women) (Table 2B). The increment in Cornell index did not differ between men and women with FD (FD: men minus women: β = 0.8 ms per decade; 95%-CI −0.1–1.7), but Cornell index was higher in men compared to women (both control subjects and patients) at all ages (Figure 2A). Compared to control subjects, FD patients had a significantly higher Cornell index from a young age onwards (from 18–29 years in men with FD and 30–39 years in women with FD) (Supplemental Table S2, Figure 4A).

Spatial QRS-T angle increased with ageing in all subgroups, but the increases were much greater in patients with FD as compared to control subjects (men: FD minus controls β = 25.1° per decade; 95%-CI 21.7–28.5, females: FD-controls: β = 24.0° per decade; 95%-CI 21.4–26.6) (Table 2B). There was overlap between the sexes in FD, both in absolute value and increment with ageing, in spatial QRS-T angle (Figure 2B). Compared to controls, both men and women with FD had a higher spatial QRS-T angle from 40 years onwards coinciding with this parameter exceeding the upper range of normal (105°) [31] (Supplemental Table S2, Figure 4B).

With ageing, the frontal QRS-axis became progressively more negative in all subgroups (i.e., left ward axis deviation), with progression being more pronounced in FD patients as compared to control subjects (men: FD minus controls: β = −6.1° per decade; CI −10.5–−1.6, women: FD minus controls: β = −4.0° per decade; CI −7.4–−0.6). There was no significant difference between men and women with FD with respect to the absolute value or change in QRS-axis deviation (Table 2B, Figure 2C). The absolute values of the frontal QRS-axis tended to be horizontal in patients with FD compared to a more normal QRS-axis in controls, but nonetheless remained within normal limits (Figure 4C).

#### 3.2.4. Frontal T-Axis

The majority of control subjects remained within the normal range of 15°−75° regardless of age [32]. However, patients with FD developed a divergent frontal T-axis from approximately 30 years (men) and 40 years (women) onwards (Supplemental Figure S5).

### 3.3. Electrocardiographic and CMR Imaging Properties in FD Patients

For the 133 included FD patients, a total of 119 CMRs (90%) were available and could be linked to the last obtained ECG during follow-up. LVMi and LGE data were reported for 101 (76%) and 118 (99%) of the 119 CMRs, respectively. See Table 1 for detailed descriptive statistics on LVMi and LGE. Based on the Spearman analyses, we found statistically significant correlations between the seven main ECG parameters (P-wave duration, PR-interval, QRS-duration, QTc, Cornell index, spatial QRS-T angle and frontal QRS axis) and LVMi (Supplemental Figure S8). The absolute values of P-wave duration, QRS-duration, QTc, Cornell index and spatial QRS-T angle were significantly divergent in patients with LGE vs. patients without LGE. The ECG parameter that best distinguishes patients with and without fibrosis was the spatial QRS-T angle, where 51 out of 53 FD patients (96%) without LGE had a normal QRS-T angle (between 0° and 105°) (Supplemental Figure S9).

## 4. Discussion

This is the first long-term longitudinal study in classical FD patients that assessed the evolution of electrophysiological depolarisation, repolarisation and their interaction. In addition, we could include ECGs from a large cross-sectional sample of apparently healthy control subjects, enabling a comparison across a wide age range.

The results show that for the studied ECG parameters, the differences between FD patients and controls increase with ageing. These parameters differ from the included control cohort, both in terms of rate of progression as well as absolute values, but they often fall within generally accepted reference ranges and may therefore not be recognized as abnormal. This is particularly true for the PR-interval and frontal QRS-axis. PR-interval has long been considered a hallmark of FD [33], but both in this study, as well as the study by Namdar et al., the absolute values of PR-intervals for most FD patients fall within the reference range (120–200 ms) [30,34].

Perhaps the most important result of this study is that, for all the ECG parameters studied, it is not so much the absolute value, but the rate of change over time that clearly distinguishes the FD patients (especially at younger age) from the control subjects (Figure 1, Figure 2, Figure 3 and Figure 4). This likely represents progression of cardiac disease since: (a) The course is very different from that of controls with cardiovascular risk factors, (b) there is a clear association between the ECG parameters and other established markers of cardiac disease (left ventricular hypertrophy and the presence of fibrosis as assessed by CMR) and (c) in studies in both the general population [31,35,36,37] and patients with other cardiac disorders [38,39,40] abnormal values of ECG parameters are known to be associated with a higher risk of cardiac complications.

Considering the described differences in the rate of change between FD patients and controls from the general population, we suggest that monitoring the rate of change in FD patients with still apparently ‘normal’ ECGs might be a suitable way, in combination with echocardiography, CMR and biochemical markers, to detect early signs of cardiac involvement in FD.

Surprisingly, for several parameters the rate of change in female patients was comparable and sometimes even more pronounced compared to male patients (e.g., P-wave duration, PR-interval, QRS- duration, Cornell index, spatial QRS-T angle and frontal QRS-axis) (Figure 1 and Figure 2). Since not all female patients will develop cardiac events of FD, it is especially important to be able to detect development of cardiac disease in these patients. As can be deducted from Supplemental Figures S2–S5 changes in ECG parameters do not occur in all patients and a proportion of the female patients follows a trajectory over time that is similar to control subjects. This suggests that these parameters may indeed be suitable to differentiate between those patients who develop progressive cardiac disease and those who do not.

The parameter that best distinguishes patients with from those without LGE is the spatial QRS-T angle, which is likely to be a much more reproducible measurement compared to other ECG parameters [41] to quantify the interaction between ventricular depolarisation and repolarisation. This study shows, for the first time, the increment in spatial QRS-T angle in FD patients. Previous studies have shown that this vectorcardiographic parameter can independently predict cardiac events in the general population and patients with other heart disease (e.g., heart failure and myocardial infarction) [31,39,40]. More longitudinal studies are required to investigate whether the spatial QRS-T angle is a useful parameter to predict cardiovascular events in FD patients.

What is needed to firmly establish these parameters as prognostic biomarkers is an established link between early ECG changes and clinical outcomes. This study shows that men with classical FD showed differences in ECG parameters compared to control subjects from age 20 years onwards, with the Cornell index as the earliest marker to change. In women with classical FD, this occurs from age 30 years onwards (Figure 4A). Whether these ECG alterations predict cardiac complications or the occurrence of LVH and LGE, that are known to occur approximately 20 years later [14,20] could not be determined because of: a) The relatively small number of cardiac events in this cohort and the absence of cardiac events in younger patients and b) the missing long-term ECG data prior to an event for patients who were diagnosed after their first cardiovascular event (especially women without a positive FD family history or classical FD symptoms). To investigate the predictive utility of ECG markers in FD patients, it is essential to conduct multicenter studies that can provide these missing data. The current study provides insight into which parameters can be studied to assess their predictive value for the development of cardiovascular complications in FD patients.

This study has several limitations. First, survival (premature death) and treatment bias (exclusion of ECGs of patients with externally paced rhythm) are attributed to selection bias. This is particularly relevant for older men with classical FD, since it has led to under-recording of ECGs of these severely affected patients. The unavoidable selection of ECGs from patients over the age of 50 with a relatively mild phenotype (severely affected patients have by then died or received a pacemaker) could incorrectly suggest that with advanced age male patients have a similar electrocardiographic phenotype as female FD patients. Second, in FD patients, changes in electrophysiological parameters reflect time-dependent alterations of each parameter (longitudinally collected ECGs). However, for the control subjects, the data were obtained in a cross-sectional study and no changes within the included subjects can be analyzed. Third, the gross majority of the included patients were treated with enzyme replacement therapy (ERT) for at least part of the follow-up and the electrophysiological changes depicted do not represent the natural disease course. This effect of treatment (ERT) on the electrophysiological parameters cannot be studied given the small untreated patient sample, the treatment indication bias and the vast difference in age at which treatment was started between patients. Previous studies indicated that cardiac disease may progress despite ERT [19,42], and this is confirmed by the current study. The data from the current study, can, however, be used to analyze the effect of new FD therapies to see if they outperform current treatment in preventing the progression of cardiac disease.

## 5. Conclusions

In FD, several ECG parameters show progressive alterations during adult life. The frontal QRS-axis is already significantly different in both male and female FD patients aged 18–29 years and is thus the earliest marker of cardiac disease identified in this study. For male patients this is also true for the Cornell index, with female patients following a decade later. For the other ECG parameters, specifically the rate of change throughout adulthood, more than the absolute values, is grossly different in FD patients from that in apparently healthy individuals. Tracking the rate of change could be a good way to detect disease progression in early adulthood, guiding treatment initiation in those that show significant changes. At later disease stages the absolute values for all ECG parameters, except for PR-interval, become abnormal, in comparison to both the controls and the reference values. All studied ECG parameters showed a positive correlation with left ventricular mass index on CMR. P-wave duration, QRS-duration, QTc, Cornell index and spatial QRS-T angle were different in patients with cardiac fibrosis compared to those without and the spatial QRS-T angle could be used to identify those requiting CMR follow up of fibrosis development.

## Figures and Tables

**Table 1:** Subjects’ characteristics.

Fabry Patients’ Characteristics			
Number of patients, n (%)	All 133 (100%)	Men 48 (36%)	Women 85 (64%)
General			
Number of ECGs	1995	819	1176
Number of ECGs per patient, median (range)	15 (4–66)	18 (4–66)	15 (4–47)
Age at first ECG (years), median (range)	38 (18–68)	27 (18–58)	41 (18–68)
Age at last ECG (years), median (range)	48 (19–82)	37 (19–65)	50 (20–82)
Follow-up duration (years), median (range)	9 (0–18)	8 (0–18)	9 (0–17)
Enzyme replacement therapy (ERT)			
Patients on ERT, n (%)	106 (80%)	45 (94%)	61 (72%)
Duration of ERT treatment (years), median (range)	7 (0–17)	9 (0–17)	7 (0–14)
Cardiovascular comorbidities			
Smoking †, n (%)	47 (35%)	16 (33%)	31 (37%)
Hypertension †, n (%)	22 (17%)	7 (15%)	15 (18%)
Obesity †, n (%)	15 (11%)	0 (0%)	15 (18%)
Diabetes mellitus †, n (%)	2 (1.5%)	0 (0%)	2 (2%)
Dyslipidemia †, n (%)	6 (5%)	0 (0%)	6 (7%)
Laboratory findings at last ECG			
eGFR- CKD EPI formula (mL/min), median (range)	87 (15–144)	101 (15–144)	85 (22–131)
Urine albumin (mg/24 h), median (range)	42 (3–3498)	54 (4–3498)	42 (3–1175)
Presence of microalbuminuria **, n (%)	65/115 (57%)	25/40 (63%)	40/75 (53%)
Cardiac MRI findings at last ECG			
Left ventricular mass index (g/m^2^), median (range)	63 (32–141)	78 (43–141)	60 (32–117)
Presence of late Gadolinium enhancement, n (%)	60/118 (51%)	17/40 (43%)	43/78 (55%)
Cardiovascular events during follow-up			
Major adverse Cardiovascular events, n (%)	22 (17%)	11 (23%)	11 (13%)
Age at MACE (years), median (range)	55 (33–70)	55 (33–66)	66 (34–70)
Controls’ characteristics			
Number of controls, n (%)	All 3893 (100%)	Men 1667 (43%)	Women 2226 (57%)
General			
Age at ECG, median (range)	46 (18–71)	45 (18–65)	46 (18–71)
Cardiovascular comorbidities			
Smoking *, n (%)	2377 (61%)	1049 (63%)	1328 (60%)
Hypertension *, n (%)	983 (25%)	514 (31%)	469 (21%)
Obesity, n (%)	345 (9%)	139 (8%)	206 (9%)
Diabetes mellitus, n (%)	102 (3%)	57 (3%)	45 (2%)
Antilipaemics, n (%)	164 (4%)	76 (5%)	88 (4%)
Laboratory findings			
eGFR- CKD EPI formula (mL/min), median (range)	97 (24–146)	99 (25–146)	95 (24–137)
Presence of microalbuminuria **, n (%)	162/3879 (4%)	69/1660 (4%)	93/2219 (4%)

† cardiovascular risk factors were for FD patients assessed at first outpatient clinic visit: -Obesity: Body Mass Index ≥ 30 kg/m^2^; -Smoking: ever smoked.; -Hypertension: antihypertensive medication use or systolic blood pressure of >140 mmHg and/or diastolic blood pressure of >90 mmHg, measured at least twice.; -Dyslipidemia: elevated levels of total cholesterol (>6.5 mmol/L) or low density lipoprotein (LDL) cholesterol (>2.5 mmol/L) or triglycerides (>3.0 mmol/L), or low levels of high-density lipoprotein (HDL) cholesterol (men: <1.0 mmol/L, women <1.2 mmol/L), or medication prescribed for the indication dyslipidemia.; -Diabetes mellitus: type I or type II if reported by a medical doctor in the medical chart or when the patient is using anti-diabetic medication. * The prevalence of smoking and hypertension in the control group was higher than in FD patients. ** Microalbuminuria in FD patients was defined as ≥30 mg albuminuria in the collected 24 h urine sample. In controls, microalbuminuria was defined as ≥20 mg/L albumin in a urine portion.

**Table 2:** Estimated regression coefficients (β) per 10 years increase in age.

**(A)**				
**Participants Subgroup**	**P-wave Duration (ms)**	**PR-Interval (ms)**	**QRS-Duration (ms)**	**QTc (ms)**
FD men	2.9 (1.7–4.2) ***	2.3 (−1.0–5.5)	10.5 (8.9–12.0) ***	20.8 (18.1–23.5) ***
Controls- men	3.2 (2.7–3.6) ***	3.6 (2.7–4.5) ***	−0.6 (−1.1–−0.2) **	4.4 (3.4–5.3) ***
FD women	5.0 (4.0–5.9) ***	10.4 (7.9–12.9) ***	9.3 (8.0–10.5) ***	12.6 (10.5–14.8) ***
Controls- women	2.6 (2.2–2.9) ***	3.3 (2.6–4.0) ***	0.3 (−0.1–0.7)	4.4 (3.7–5.1) ***
**Estimated differences in regression coefficients (β) per 10 years increase in age between study participants subgroups with 95%-CI**				
FD men minus FD women	−2.1 (−3.6–−0.5) *	−8.2 (−12.3–−4.1) ***	1.2 (−0.8–3.2)	8.2 (4.7–11.6) ***
FD men minus Controls- men	−0.2 (−1.6–1.1)	−1.3 (−4.7–−2.1)	11.1 (9.4–12.7) ***	16.4 (13.6–19.3) ***
FD women minus Controls- women	2.4 (1.4–3.4) ***	7.1 (4.5–9.7) ***	9.0 (7.7–10.2) ***	8.2 (6.0–10.5) ***
Controls- men minus Controls- women	0.6 (0.001–1.1) *	0.3 (−0.9–1.4)	−0.9 (−0.15–−0.3) **	0.0 (−0.11–0.12)
**(B)**				
**Other LV ECG parameters- estimated regression coefficients (β) per 10 years increase in age in each study participants subgroup with 95%-CI (based on the GLM)** * *p* < 0.05, ** *p* < 0.01, *** *p* < 0.001				
**Participants Subgroup**	**Cornell Index (mm)**	**Spatial QRS-T Angle (°)**	**Frontal QRS-Axis (°)**	
FD men	2.6 (1.9−3.4) ***	29.4 (26.1–32.6) ***	−15.5 (−19.7–−11.2) ***	
Controls- men	0.0 (−0.2–0.3)	4.3 (3.3–5.3) ***	−9.4 (−10.7–−8.1) ***	
FD women	1.8 (1.3–2.4) ***	27.7 (25.2–30.2) ***	−11.4 (−14.6–−8.2) ***	
Controls- women	0.6 (0.4–0.8) ***	3.7 (2.9–4.5) ***	−7.4 (−8.4–−6.3) ***	
**Estimated differences in regression coefficients (β) per 10 years increase in age between study participants subgroups with 95%-CI**				
FD men minus FD women	0.8 (−0.1–1.7)	1.7 (−2.4–5.8)	−4.1 (−9.4–1.3)	
FD men minus Controls- men	2.6 (1.8–3.4) ***	25.1 (21.7–28.5) ***	−6.1 (−10.5–−1.6) **	
FD women minus Controls- women	1.2 (0.7–1.8) ***	24.0 (21.4–26.6) ***	−4.0 (−7.4–−0.6) *	
Controls- men minus Controls- women	0.6 (0.3–0.9) ***	0.6 (0.7–1.9)	−2.0 (−3.7–−0.4) *	

## Data Availability

The data presented in this study are available on request from the corresponding author. The data are not publicly available due to the rarity of the disease, even anonymized data can be linked to a specific individual. In case of a specific scientific question, requests to make parts of the data set available will be reviewed.

## Supplementary Materials

The following supporting information can be downloaded at: https://www.mdpi.com/article/10.3390/diagnostics13030354/s1.
