# Supplementary material for: ECG Changes during Adult Life in Fabry Disease: Results from a Large Longitudinal Cohort Study
Source: Diagnostics (Basel). 2023 Jan 18;13(3):354. doi: 10.3390/diagnostics13030354 (PMC9913957; doi:10.3390/diagnostics13030354)
Supplement: Supplementary file 1 [file diagnostics-13-00354-s001.zip › diagnostics-2062557-supplementary.pdf]

SUPPLEMENTAL MATERIAL

Supplemental table 1A: Generalized linear mixed model of the conduction times												
	P-wave duration (ms)			PR-interval (ms)			QRS-duration (ms)			QTc (ms)		
Model 1 (age, Type and Sex separated)												
**Fixed effects**	β (95% CI)		p value	β (95% CI)		p value	β (95% CI)		p value	β (95% CI)		p value
Age	0.28	0.25 – 0.31	<0.001	0.38	0.32 – 0.43	<0.001	0.05	0.02 – 0.09	0.001	0.54	0.49 – 0.60	<0.001
Type (FD patient)	1.40	0.02 – 2.77	0.046	-14.65	-18.00 – -11.30	<0.001	2.92	1.35 – 4.48	<0.001	-1.18	-3.82 – 1.46	0.381
Sex (Male)	6.94	6.21 – 7.67	<0.001	8.54	7.09 –9.99	<0.001	9.93	9.23 – 10.62	<0.001	-8.84	-10.33 – -7.36	<0.001
Model 2 (Controls and FD patients combined groups- with age*Type*Sex interactions)												
(Intercept)	96.62	95.00 – 98.23		140.30	136.94 – 143.66		90.91	89.30 – 92.53		401.60	398.23 – 404.97	
Age	0.26	0.22 – 0.29	<0.001	0.33	0.26 – 0.40	<0.001	0.03	-0.01 – 0.07	0.114	0.44	0.37 – 0.51	<0.001
Type (FD patient)	-7.40	-12.02 – -2.77	0.002	-43.83	-55.51 – -32.14	<0.001	-34.51	-39.91 – -29.12	<0.001	-38.99	-49.24 – -28.74	<0.001
Sex (Male)	4.55	1.95 – 7.15	0.001	7.10	1.75 – 12.46	0.009	13.27	10.70 – 15.83	<0.001	-9.09	-14.48 – -3.70	0.001
**Interactions**												
Age*Type (FD patient)	0.24	0.14 – 0.34	<0.001	0.71	0.45 – 0.97	<0.001	0.90	0.77 – 1.02	<0.001	0.82	0.60 – 1.05	<0.001
Age*Sex (Male)	0.06	0.00 – 0.11	0.045	0.03	-0.09 – 0.14	0.653	-0.09	-0.15 – -0.03	0.002	-0.01	-0.12 – 0.11	0.904
Type (FD patient)*Sex (Male)	11.44	4.29 – 18.59	0.002	39.40	21.08 – 57.71	<0.001	0.89	-7.56 – 9.34	0.837	-20.13	-36.05 – -4.20	0.013
Age*Type (FD patient)*Sex (Male)	-0.26	-0.43 – -0.10	0.002	-0.84	-1.27 – -0.42	<0.001	0.21	0.00 – 0.42	0.049	0.82	0.46 – 1.19	<0.001

Supplemental table 1B: Generalized linear mixed model of the other LV related ECG parameters									
	Cornell index (mm)			Spatial QRS-T angle (°)			Frontal QRS-axis (°)		
Model 1 (age, Type and Sex separated)									
**Fixed effects**	β (95% CI)		p value	β (95% CI)		p value	β (95% CI)		p value
Age	0.04	0.02 – 0.05	<0.001	0.53	0.47 – 0.60	<0.001	-0.84	-0.92 – -0.77	<0.001
Type (FD patient)	8.22	7.40 – 9.03	<0.001	35.91	32.10 – 39.73	<0.001	-6.82	-12.06 – -1.58	0.011
Sex (Male)	3.39	2.98 – 3.80	<0.001	5.07	3.33 – 6.80	<0.001	-3.93	-6.05 – -1.80	<0.001
Model 2 (Controls and FD patients combined groups- with age*Type*Sex interactions)									
(Intercept)	7.94	7.05 – 8.82		28.73	25.17 – 32.30		77.72	72.99 – 82.44	
Age	0.06	0.04 – 0.08	<0.001	0.37	0.29 – 0.45	<0.001	-0.74	-0.84 – -0.63	<0.001
Type (FD patient)	2.87	0.22 – 5.51	0.034	-57.61	-69.01 – -46.21	<0.001	5.00	-10.16 – 20.16	0.518
Sex (Male)	5.95	4.54 – 7.37	<0.001	3.63	-2.08 – 9.34	0.213	4.42	-3.11 – 11.96	0.250
Age*Type (FD patient)	0.12	0.07 – 0.18	<0.001	2.40	2.14 – 2.66	<0.001	-0.40	-0.74 – -0.06	0.020
Age*Sex (Male)	-0.06	-0.09 – -0.03	<0.001	0.06	-0.07 – 0.19	0.344	-0.20	-0.37 – -0.04	0.017
Type (FD patient)*Sex (Male)	-4.25	-8.33 – -0.17	0.041	-8.01	-25.69 – 9.68	0.375	8.42	-15.54 – 32.39	0.491
Age*Type (FD patient)*Sex (Male)	0.14	0.04 – 0.23	0.005	0.11	-0.32 – 0.54	0.619	-0.20	-0.76 – 0.36	0.477

**Supplemental table 1C**				
**Conduction times - estimated regression coefficients (β) per 10 years increase in age in ERT treated versus ERT treated and untreated FD patients with 95%-CI (based on the GLM)** **** p< 0.05, ** p< 0.01, ***p<0.001***				
**ERT treated**	**P-wave duration (ms)**	**PR-interval (ms)**	**QRS-duration (ms)**	**QTc (ms)**
FD men	**2.7 (1.5-4.0)*****	2.1 (-1.2-5.4)	**10.9 (9.3-12.4)*****	**20.8 (18.0-23.5)*****
FD women	**5.3 (4.2-6.4)*****	**12.4 (9.5-15.3)*****	**10.1 (8.7- 11.5)*****	**13.8 (11.3-16.2)*****
**ERT treated and untreated**	**P-wave duration (ms)**	**PR-interval (ms)**	**QRS-duration (ms)**	**QTc (ms)**
FD men	**2.9 (1.7-4.2)*****	2.3 (-1.0-5.5)	**10.5 (8.9-12.0)*****	**20.8 (18.1-23.5)*****
FD women	**5.0 (4.0-5.9)*****	**10.4 (7.9-12.9)*****	**9.3 (8.0-10.5)*****	**12.6 (10.5-14.8)*****

**Supplemental table 1D**			
**Other LV ECG parameters - estimated regression coefficients (β) per 10 years increase in age in ERT treated versus ERT treated and untreated FD patients with 95%-CI (based on the GLM)** **** p< 0.05, ** p< 0.01, ***p<0.001***			
**ERT treated**	**Cornell index (mm)**	**Spatial QRS-T angle (°)**	**Frontal QRS-axis (°)**
FD men	**2.8 (2.0-3.5)*****	**29.7 (26.4-33.0)*****	**-16.2 (-20.6- -11.8)*****
FD women	**1.6 (1.0-2.2)*****	**28.5 (25.6- 31.4)*****	**-11.9 (-15.6- -8.1)*****
**ERT treated and untreated**	**Cornell index (mm)**	**Spatial QRS-T angle (°)**	**Frontal QRS-axis (°)**
FD men	**2.6 (1.9-3.4)*****	**29.4 (26.1-32.6)*****	**-15.5 (-19.7- -11.2)*****
FD women	**1.8 (1.3-2.4)*****	**27.7 (25.2-30.2)*****	**-11.4 (-14.6- -8.2)*****

Supplemental table 2: ECG descriptives per age decade (the last available ECG per FD patient per decade was selected to ensure that the influence of repeated measurements was limited).
**p<0.05, ** p<0.01, ***p<0.001*

**P-wave duration in men (ms)**						**P-wave duration in women (ms)**					
Age category	Available ECGs in Fabry men, n	Fabry men Median (IQR)	Available ECGs in Controls- men, n	Controls- men Median (IQR)	P- value	Age category	Available ECGs in Fabry women, n	Fabry women Median (IQR)	Available ECGs in Controls- women, n	Controls- women Median (IQR)	P- value
18-29	25	110 (102-118)	262	108 (102-116)	1.0	18-29	28	105 (97-111)	435	104 (96-110)	1.0
30-39	13	120 (108-122)	379	112 (106-118)	0.2	30-39	21	108 (102-114)	393	106 (98-112)	1.0
40-49	15	126 (120-128)	374	116 (110-122)	**0.01***	40-49	32	110 (102-120)	463	108 (100-114)	1.0
50-59	9	128 (114-130)	409	118 (112-124)	1.0	50-59	32	116 (110-129)	519	112 (104-118)	**0.03***
60-69	1	150 (-)	235	121 (112-128)	0.4	60-69	9	122 (116-128)	399	114 (106-120)	0.1
70+	-	-	-	-	-	70+	1	156 (-)	11	112 (102-121)	0.9
	**PR-interval in men (ms)**					**PR-interval in women (ms)**					
Age category	Available ECGs in Fabry men, n	Fabry men Median (IQR)	Available ECGs in Controls- men, n	Controls- men Median (IQR)	P- value	Age category	Available ECGs in Fabry women, n	Fabry women Median (IQR)	Available ECGs in Controls- women, n	Controls- women Median (IQR)	P- value
18-29	25	146 (138-158)	262	156 (142-170)	0.6	18-29	28	132 (124-143)	435	146 (134-160)	**0.004****
30-39	13	152 (132-176)	379	158 (144-174)	1.0	30-39	21	134 (126-162)	393	152 (140-164)	0.1
40-49	15	144 (134-149)	374	162 (150-176)	**0.02***	40-49	32	140 (122-155)	463	152 (140-166)	**0.01***
50-59	9	146 (132-162)	409	166 (152-178)	0.1	50-59	32	158 (132-177)	519	158 (144-172)	1.0
60-69	1	186 (-)	235	168 (152-188)	1.0	60-69	9	180 (170-184)	399	158 (146-174)	0.1
70+	-	-	-	-	-	70+	1	244 (-)	11	166 (146-174)	0.9
	**QRS-duration in men (ms)**					**QRS-duration in women (ms)**					
Age category	Available ECGs in Fabry men, n	Fabry men Median (IQR)	Available ECGs in Controls- men, n	Controls- men Median (IQR)	P- value	Age category	Available ECGs in Fabry women, n	Fabry women Median (IQR)	Available ECGs in Controls- women, n	Controls- women Median (IQR)	P- value
18-29	25	104 (94-108)	262	102 (96-108)	1.0	18-29	28	87 (84-94)	435	92 (86-98)	0.06
30-39	13	102 (96-114)	379	102 (96-108)	1.0	30-39	21	96 (86-100)	393	90 (86-96)	1.0
40-49	18	113 (105-137)	374	102 (96-106)	**<0.001*****	40-49	34	97 (88-106)	463	90 (86-97)	**0.02***
50-59	15	134 (121-149)	415	100 (94-106)	**<0.001*****	50-59	38	108 (99-116)	519	92 (86-98)	**<0.001*****
60-69	3	166 (159-184)	237	100 (94-106)	**0.02***	60-69	16	117 (96-145)	405	92 (86-98)	**<0.001*****
70+	-	-	-	-	-	70+	4	152 (136-173)	11	90 (85-98)	**0.04***
	**QTc in men (ms)**					**QTc in women (ms)**					
Age category	Available ECGs in Fabry men, n	Fabry men Median (IQR)	Available ECGs in Controls- men, n	Controls- men Median (IQR)	P- value	Age category	Available ECGs in Fabry women, n	Fabry women Median (IQR)	Available ECGs in Controls- women, n	Controls- women Median (IQR)	P- value
18-29	25	388 (373-398)	262	405 (387-418)	**0.02***	18-29	28	394 (385-413)	435	412 (399-424)	**<0.001*****
30-39	13	414 (410-431)	379	407 (394-422)	0.3	30-39	21	410 (396-423)	393	418 (406-432)	0.2
40-49	18	428 (420-452)	374	411 (396-424)	**0.001****	40-49	34	424 (402-432)	463	421 (409-435)	1.0
50-59	15	438 (414-478)	415	416 (402-430)	**0.02***	50-59	38	429 (417-449)	519	424 (411-437)	0.08
60-69	3	492 (475-517)	237	422 (405-436)	**0.02***	60-69	16	456 (424-485)	405	429 (417-445)	0.1
70+	-	-	-	-	-	70+	4	496 (478-527)	11	428 (419-444)	**0.009****

Cornell index in men (ms)						Cornell index in women (ms)					
Age category	Available ECGs in Fabry men, n	Fabry men Median (IQR)	Available ECGs in Controls- men, n	Controls- men Median (IQR)	P- value	Age category	Available ECGs in Fabry women, n	Fabry women Median (IQR)	Available ECGs in Controls- women, n	Controls- women Median (IQR)	P- value
18-29	25	19 (15-22)	262	14 (10-18)	**0.004****	18-29	28	12 (9-14)	435	9 (6-12)	0.3
30-39	13	17 (12-26)	379	13 (9-17)	0.1	30-39	21	14 (9-20)	393	10 (7-13)	**0.008****
40-49	18	27 (16-34)	374	13 (10-17)	**<0.001*****	40-49	34	21 (13-30)	463	9 (7-13)	**<0.001*****
50-59	15	25 (21-32)	415	14 (9-17)	**<0.001*****	50-59	38	20 (13-29)	519	10 (7-13)	**<0.001*****
60-69	3	19 (15-23)	237	14 (11-18)	1.0	60-69	16	20 (14-23)	405	13 (9-16)	**0.003****
70+	-	-	-	-	-	70+	4	13 (6-19)	11	13 (10-14)	1.0
	Spatial QRS-T angle in men (°)					Spatial QRS-T angle in women (°)					
Age category	Available ECGs in Fabry men, n	Fabry men Median (IQR)	Available ECGs in Controls- men, n	Controls- men Median (IQR)	P- value	Age category	Available ECGs in Fabry women, n	Fabry women Median (IQR)	Available ECGs in Controls- women, n	Controls- women Median (IQR)	P- value
18-29	25	33 (24-40)	262	42 (28-57)	0.09	18-29	28	29 (20-37)	435	37 (25-48)	0.1
30-39	13	37 (24-126)	379	45 (31-60)	1.0	30-39	21	31 (22-60)	393	38 (28-50)	1.0
40-49	18	137 (98-159)	374	47 (35-63)	**<0.001*****	40-49	34	148 (111-161)	463	42 (31-56)	**<0.001*****
50-59	15	148 (122-154)	415	55 (38-71)	**<0.001*****	50-59	38	154 (126-166)	519	47 (33-60)	**<0.001*****
60-69	3	149 (79-155)	237	54 (43-75)	1.0	60-69	16	151 (137-169)	405	49 (36-69)	**<0.001*****
70+	-	-	-	-	-	70+	4	157 (139-164)	11	54 (40-73)	**0.03***
	Frontal QRS-axis in men (°)					Frontal QRS-axis in women (°)					
Age category	Available ECGs in Fabry men, n	Fabry men Median (IQR)	Available ECGs in Controls- men, n	Controls- men Median (IQR)	P- value	Age category	Available ECGs in Fabry women, n	Fabry women Median (IQR)	Available ECGs in Controls- women, n	Controls- women Median (IQR)	P- value
18-29	25	50 (33-61)	262	64 (44-79)	**0.01***	18-29	28	47 (30-63)	434	64 (48-76)	**0.008****
30-39	13	41 (31-55)	378	57 (32-72)	0.3	30-39	21	37 (21-46)	393	59 (37-71)	**0.03***
40-49	18	36 (10-50)	374	47 (19-67)	0.2	40-49	34	32 (11-53)	463	55 (30-70)	**0.002****
50-59	15	7 (-25-31)	415	33 (5-60)	**0.02***	50-59	38	25 (4-46)	519	42 (18-63)	**0.01***
60-69	3	-48 (-57-25)	237	26 (-4-57)	1.0	60-69	16	21 (-4.0-42)	404	30 (8-52)	1.0
70+	-	-	-	-	-	70+	4	50 (1-92)	11	15 (7-20)	1.0

**Supplemental table 3- Definitions of cardiac events [1]**	
**Events**	**Definition**
Major adverse cardiovascular events (MACE): composite of cardiovascular death, heart failure hospitalization, sustained ventricular arrhythmias (SVA) and myocardial infarction	
1. Cardiovascular death	Death as a result of one of the following diseases/ syndromes:<br>- Acute coronary syndrome<br>- Sudden cardiac death (SCD)<br>- Hypertensive crise<br>- Ischemic or hemorrhagic stroke<br>- Cardiomyopathy<br>- Other cardiovascular cause such as: pulmonary embolism, peripheral vascular disease
2. Heart failure hospitalization	Hospital admission (at least one night) with the following clinical manifestations of heart failure: dyspnea, reduced exercise tolerance, fluid retention in peripheral and/ or splanchnic vessels, seen as peripheral edema
3. Sustained ventricular arrhythmia (SVA)	composite of sudden cardiac death (SCD), sudden cardiac arrest (SCA), sustained ventricular tachycardia (VT) including appropriate ICD shock, and ventricular fibrillation (VF)
4. Myocardial infarction	Acute myocardial injury with clinical evidence of acute myocardial ischemia. Definition according to the Fourth Universal Definition of Myocardial Infarction

1. El Sayed, M., et al., *Influence of sex and phenotype on cardiac outcomes in patients with Fabry disease.* Heart, 2021.

**Supplemental table 4: GLA mutations frequencies**		
**GLA mutation at DNA level**	Frequency in FD- men (N)	Frequency in FD- women (N)
c.1025G>A	8	13
c.1040dupT	0	2
c.1074_1075del	1	3
c.1118G>A	1	2
c.1124_1176del	2	0
c.1156C>T	2	1
c.1246C>T	1	1
c.136C>T	0	1
c.157_160delAACC	2	1
c.215T>G	1	0
c.269G>A	0	1
c.334C>T	1	1
c.400delT	0	2
c.406G>T	5	4
c.422C>T	1	1
c.53T>C	1	11
c.548G>A	1	3
c.606T>G	0	1
c.62T>C	0	3
c.639+1G>A	0	1
c.658C>T	5	4
c.677G>A	2	3
c.679C>T	2	2
c.680G>A	1	2
c.728_744del	0	4
c.779G>A	0	1
c.803T>C	2	1
c.898C>T	0	4
c.901C>T	3	2
c.908_910delTCA	0	1
c.955_969delinsTTGC	2	1
c.963_964delinsCA	1	2
c.996_999del	0	2
c.997C>T	0	1
Duplication exon 3+4	1	0
IVS6-2A>T (c.1000-2A>T)	1	2

Supplemental figures- legends

Supplemental figure 1: Flowchart for the diagnosis and phenotype allocation in FD; cerebral autosomal dominant arteriopathy with subcortical infarcts and leucoencephalopathy (CADASIL); cerebral autosomal recessive arteriopathy with subcortical infarcts and leucoencephalopathy (CARASIL); electron microscopy (EM); galactosidase alpha (GLA); diastolic interventricular septum thickness (IVSd) [1].

Supplemental figure 2: Source raw data of P-wave duration, PR-interval, QRS-duration and QTc for each study participants subgroup. The shaded areas represent the 95%-CI for the GLM fitted curves.

Supplemental figure 3: Source raw data of Cornell index (Cind), Spatial QRS-T angle and Frontal QRS-axis for each study participants subgroup. The shaded areas represent the 95%-CI for the GLM fitted curves.

Supplemental figure 4: Source raw data of the frontal T-axis for each study participants subgroup. The T-axis' normal value (15°-75°) is represented by the green lines [2].

Supplemental figure 5: Polar plot graphs showing the absolute values of the frontal T-axis for each study participants subgroup. The numbered shells represent the age at which an ECG was obtained. The T-axis' normal value (15°-75°) is represented by the green lines [2].

Supplemental figure 6: Source raw data of the heart rate for each study participants subgroup.

Supplemental figure 7: Boxplots of the heart rate per age decade in Fabry patients and controls. Numbers inside the boxes are the numbers of the analyzed ECGs. The last available ECG per FD patient per decade was selected to ensure that the influence of repeated measurements was limited. The horizontal lines represents the reference range, based on the literature.

Supplemental figure 8: Scatter plot displaying the Spearman correlation between ECG markers obtained from the last ECG and corresponding LVMi on CMR. The shaded areas represent the 95%-CI for the fitted curve.

Supplemental figure 9: Boxplots of the ECG parameters obtained from the last ECG versus the corresponding scored LGE on CMR.

References

1. El Sayed, M., et al., *Influence of sex and phenotype on cardiac outcomes in patients with Fabry disease.* Heart, 2021.
2. Kors, J.A., et al., *T axis as an indicator of risk of cardiac events in elderly people.* The Lancet, 1998. **352**(9128): p. 601-605.

Supplemental figure 1: Flowchart for the diagnosis of Fabry disease (doi:10.1136/heartjnl-2020-317922)

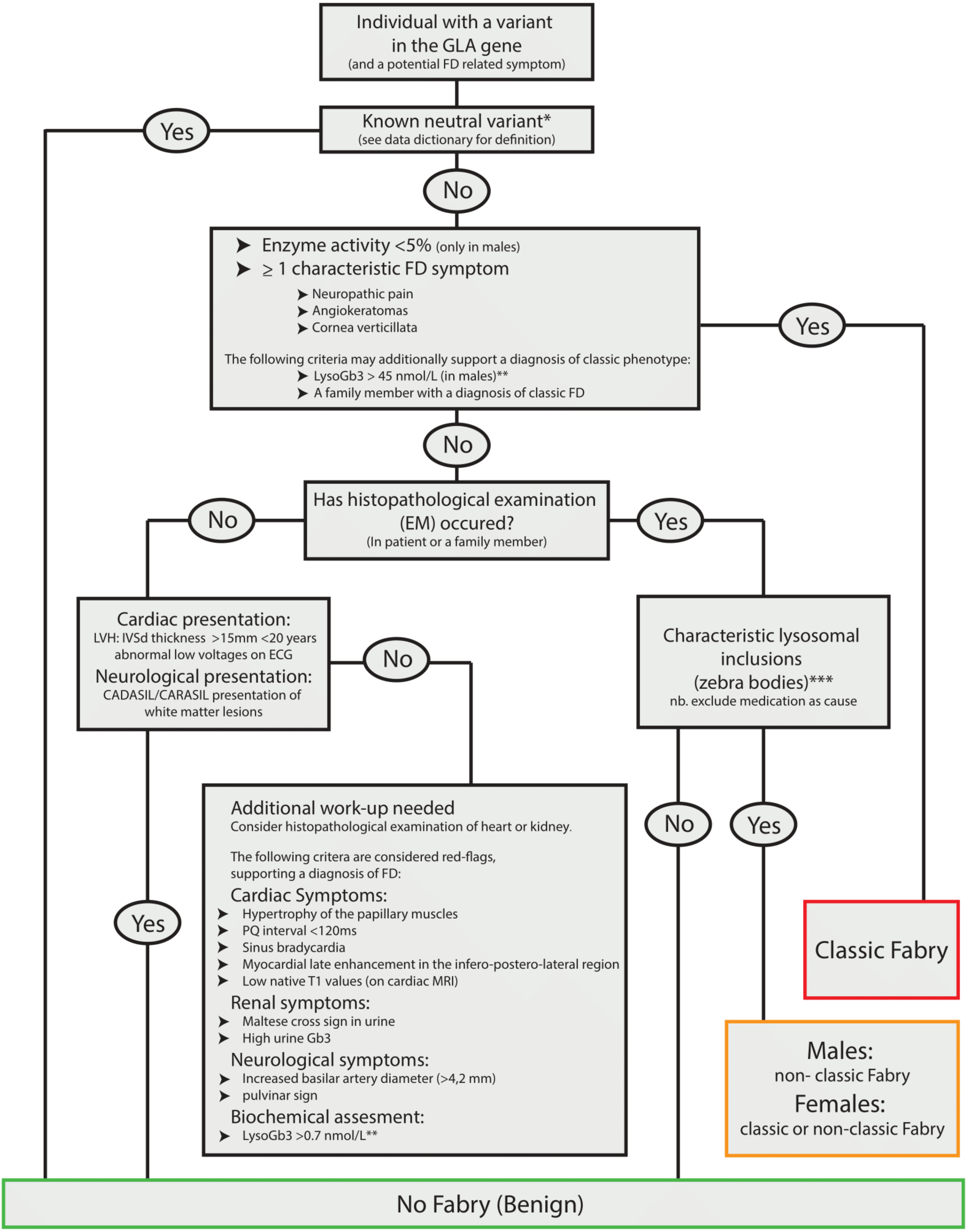

* Known neutral variants by themselves do not cause Fabry disease and thus do not explain the potential FD related symptoms. However the presence of a second, disease causing, variant in the same patient, though very rare, can occur. In case of high clinical suspicion, performing additional diagnostics (lysoGb3 levels, enzyme activity measurement) can thus be considered.

** Plasma lysoGb3 levels may help phenotypic classification (Smid et al 2015, reference values GMD laboratory Amsterdam).

*** Concentric multi-lamellated myelin bodies with a zebra like pattern and a periodicity of aproximately 5nm (Takahashi et al 1987)

This flow-chart is based on several diagnostic algorithms developed by experts. References: Smid et al 2014, van der Tol et al 2014, van der Tol et al 2015.

Supplemental figure 2

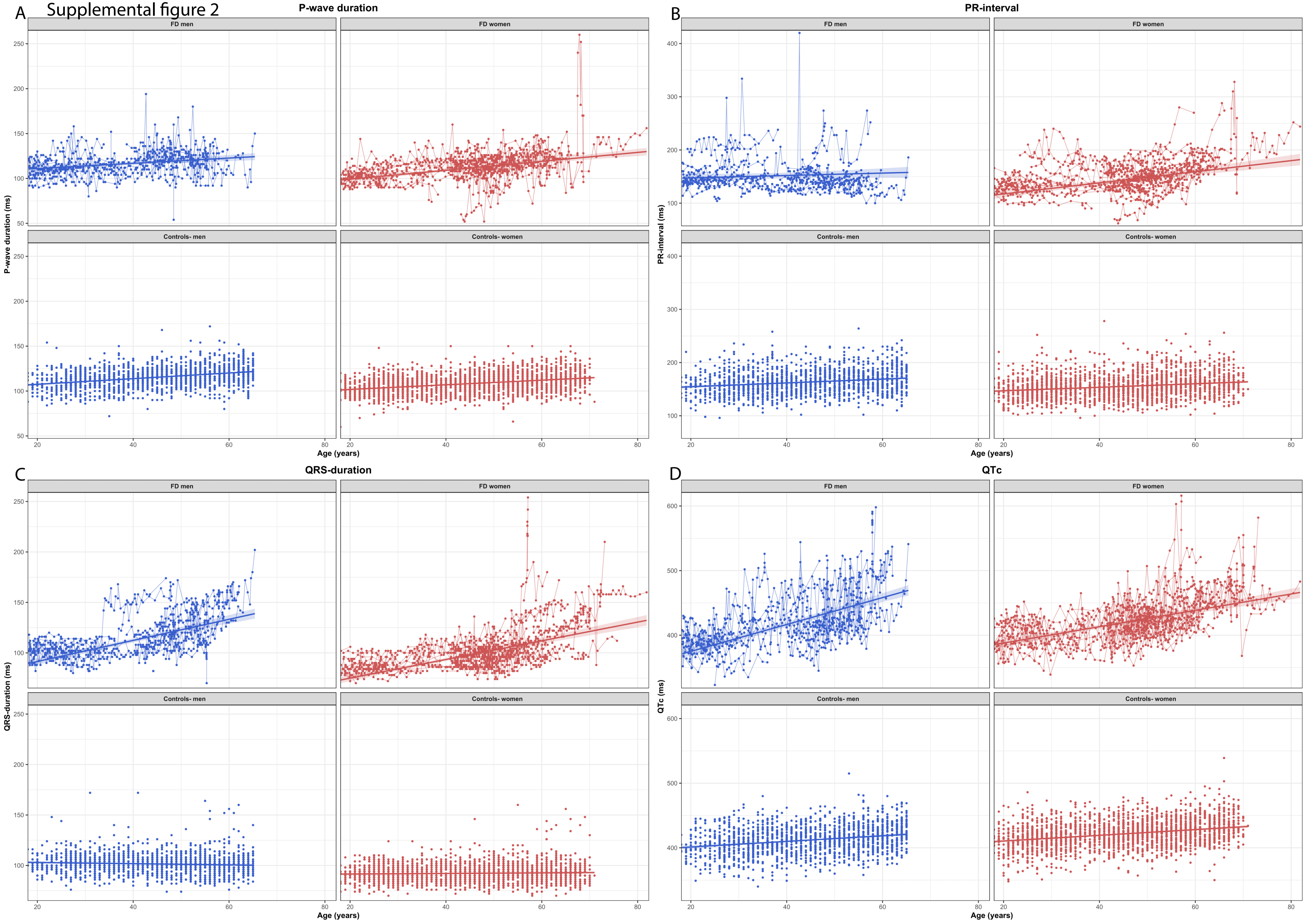

Supplemental figure 3

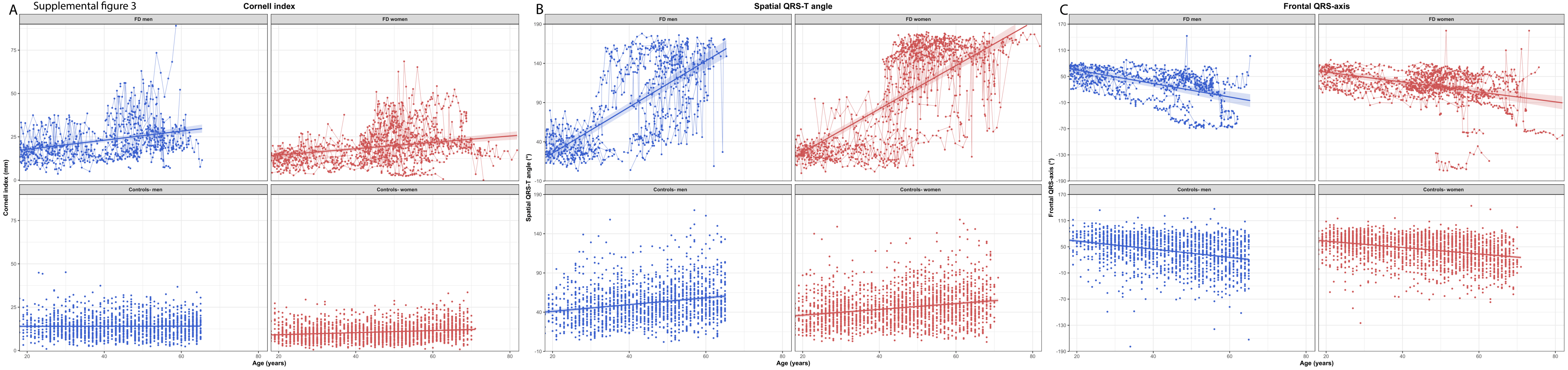

Supplemental figure 4

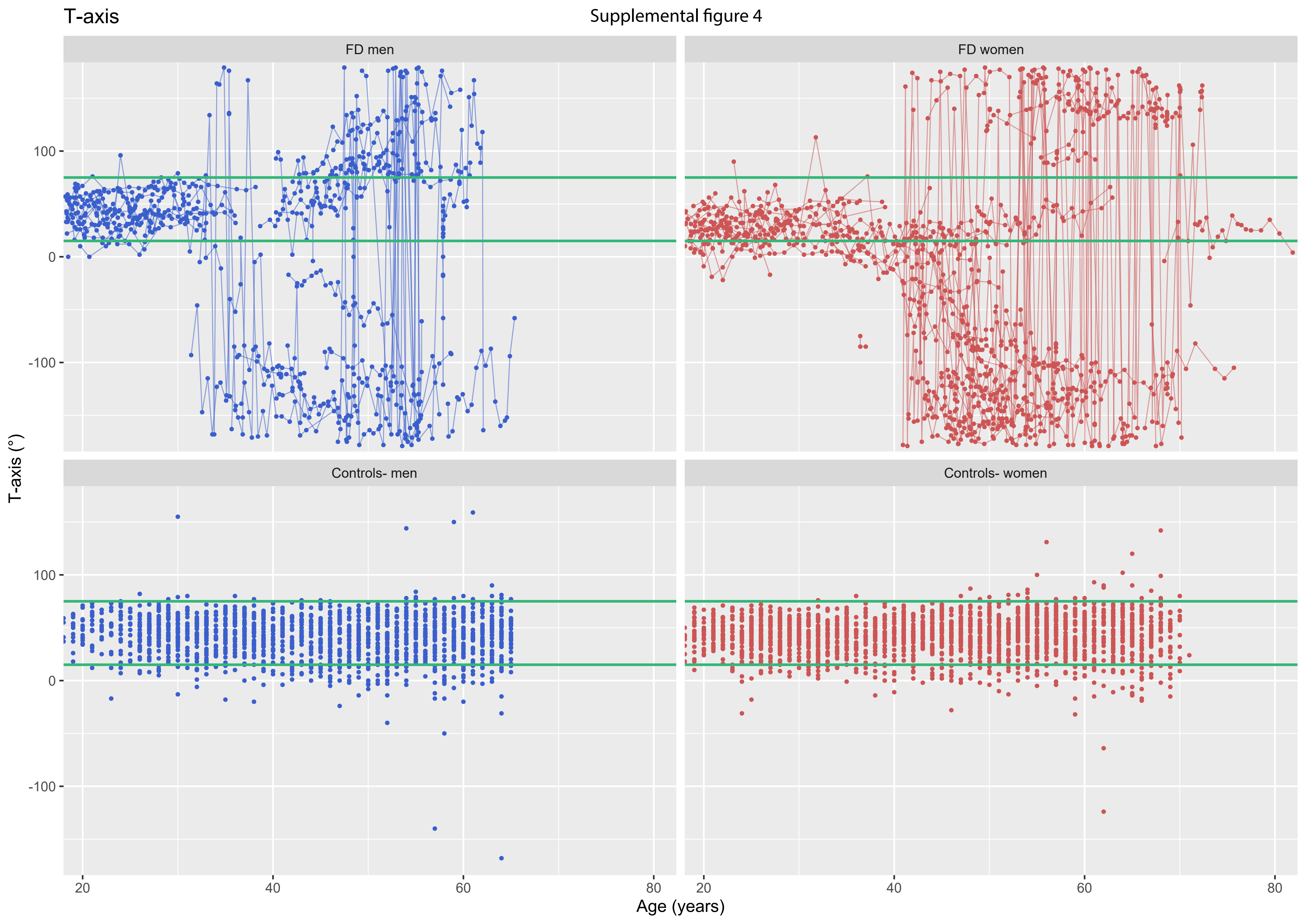

Supplemental figure 5

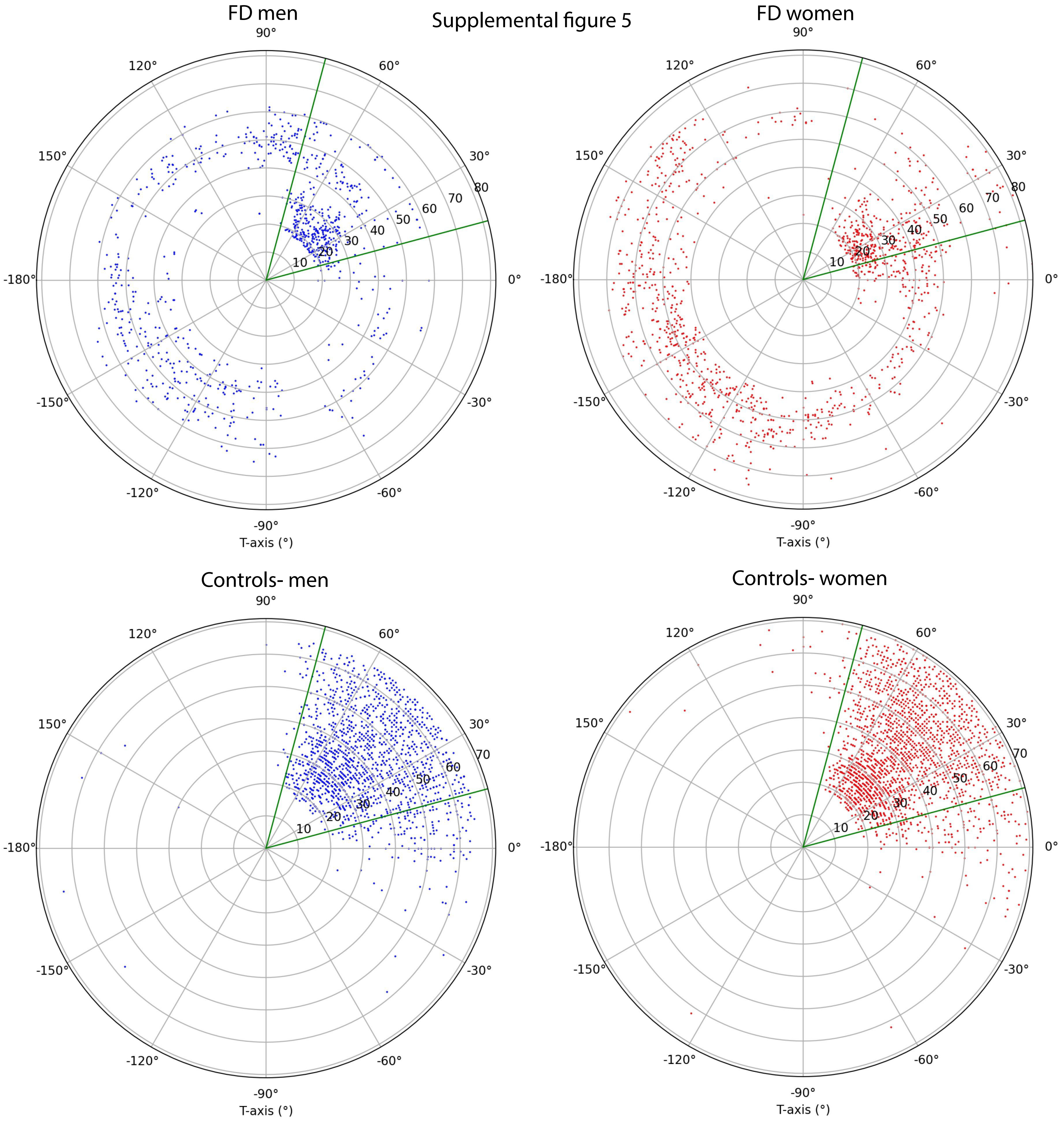

Supplemental figure 6

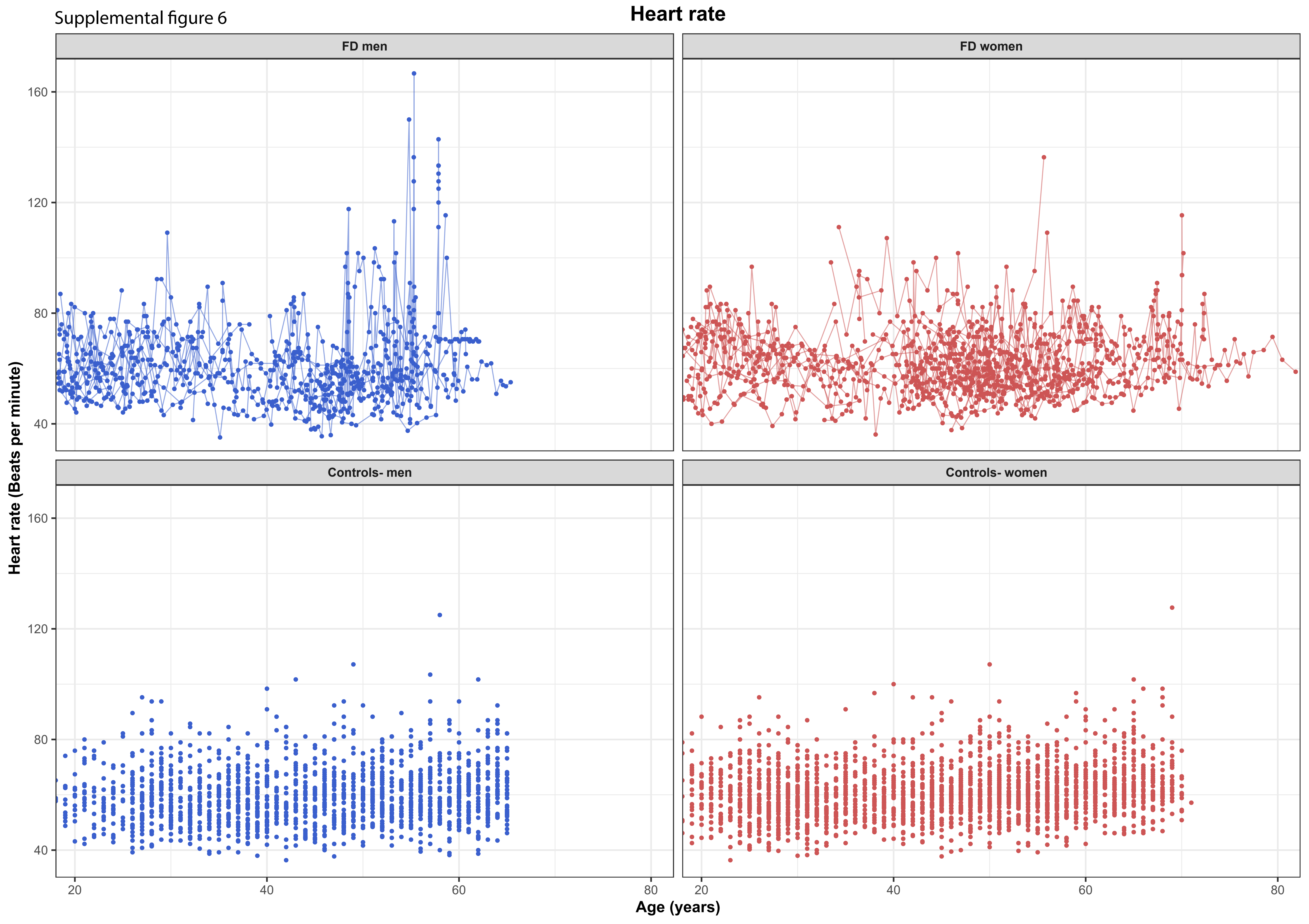

Supplemental figure 7

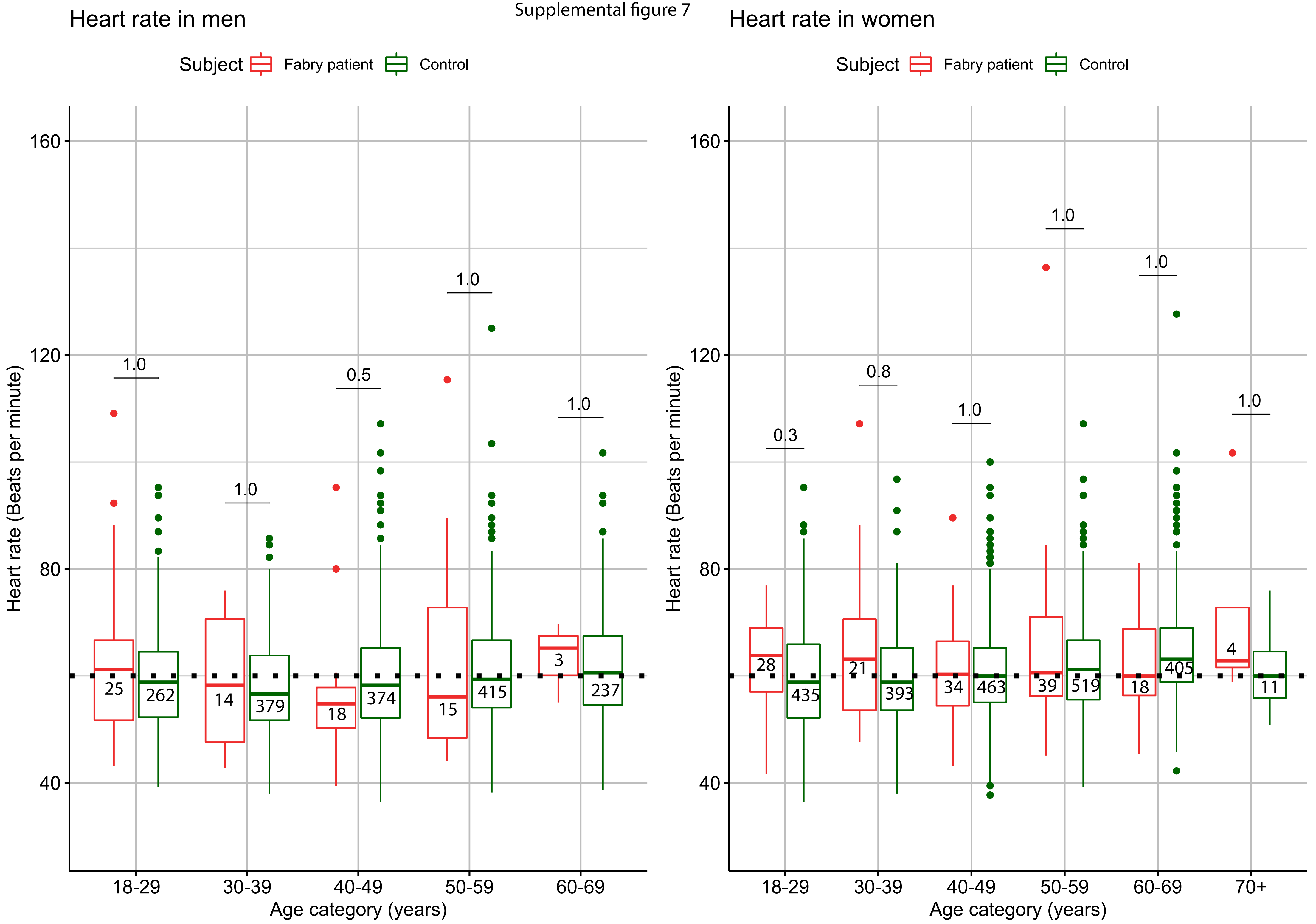

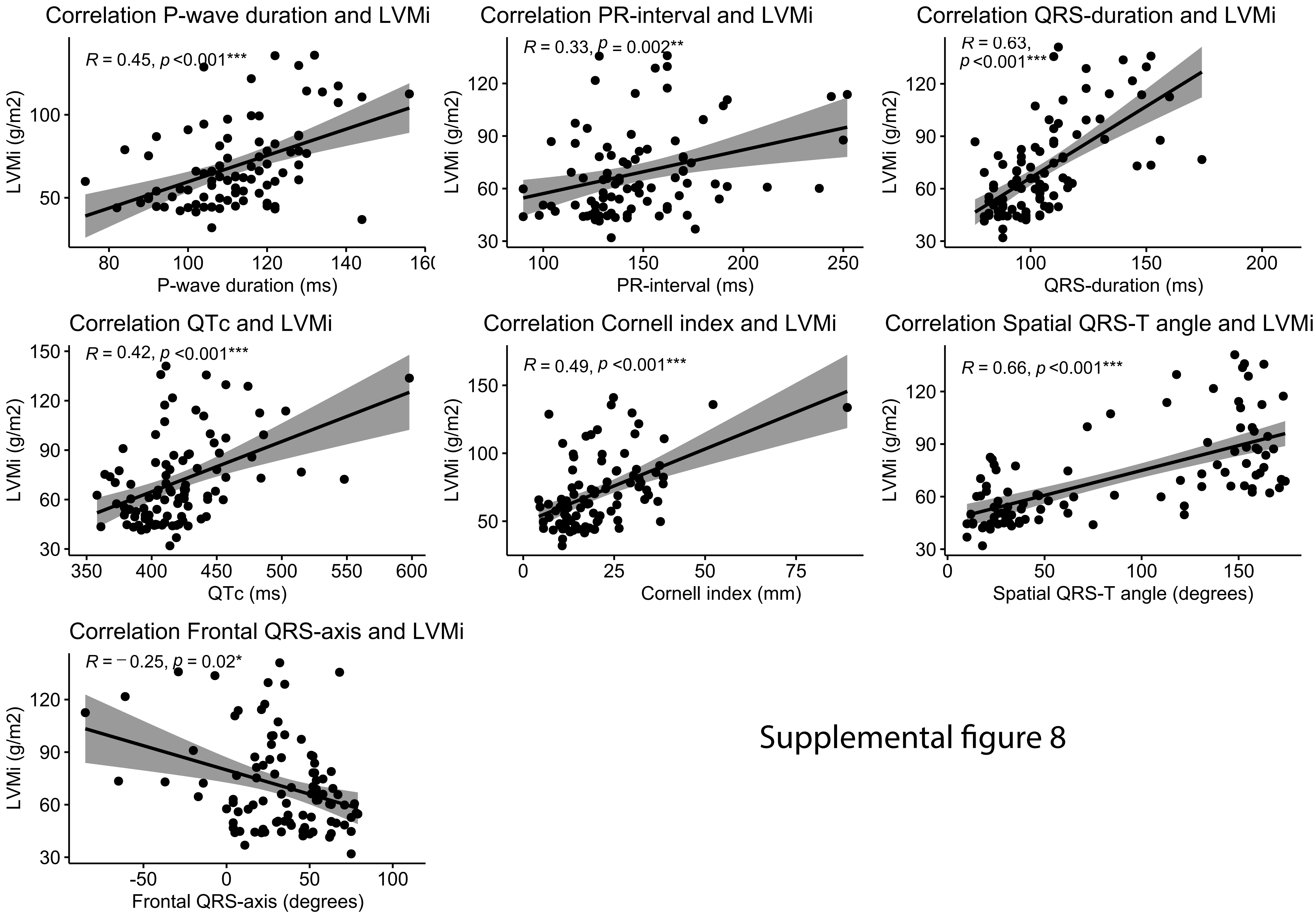

Supplemental figure 8

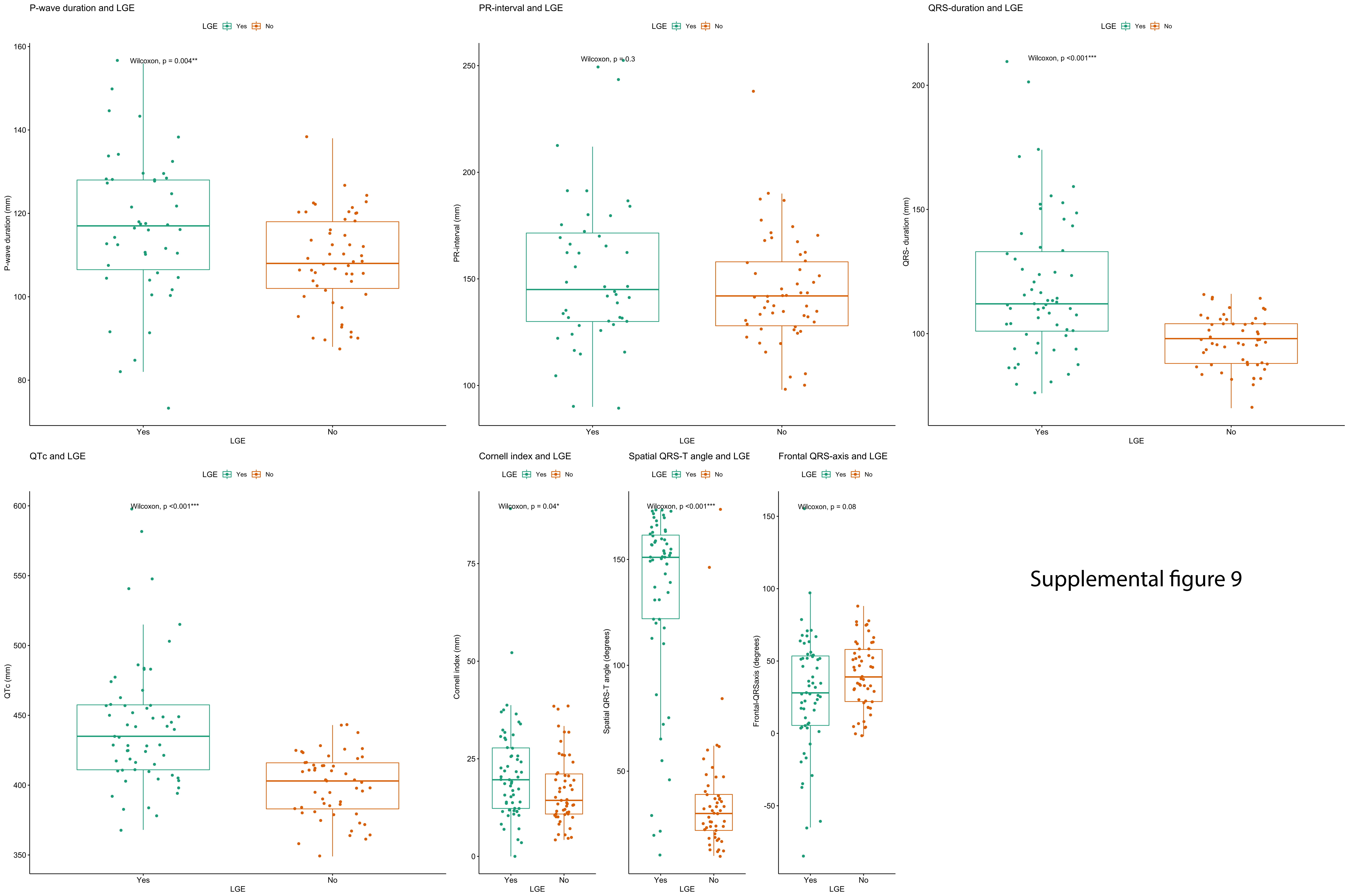

Supplemental figure 9
